# The connection between *Trypanosoma cruzi* transmission cycles by *Triatoma brasiliensis brasiliensis*: A threat to human health in an area susceptible to desertification in the Seridó, Rio Grande do Norte, Brazil

**DOI:** 10.1371/journal.pntd.0009919

**Published:** 2021-11-09

**Authors:** Vanessa Lima-Neiva, Helena Keiko Toma, Lúcia Maria Abrantes Aguiar, Catarina Macedo Lopes, Letícia Paschoaletto Dias, Teresa Cristina Monte Gonçalves, Jane Costa

**Affiliations:** 1 Laboratório de Biodiversidade Entomológica, Instituto Oswaldo Cruz/FIOCRUZ, Rio de Janeiro, Rio de Janeiro, Brazil; 2 Laboratório de Diagnóstico Molecular e Hematologia, Faculdade de Farmácia, Universidade Federal do Rio de Janeiro, Rio de Janeiro, Rio de Janeiro, Brazil; 3 Secretaria de Estado da Saúde Pública, Natal, Rio Grande do Norte, Brazil; 4 Laboratório Interdisciplinar de Vigilância Entomológica em Diptera e Hemiptera, Instituto Oswaldo Cruz /FIOCRUZ, Rio de Janeiro, Rio de Janeiro, Brazil; RTI International, UNITED STATES

## Abstract

An outbreak of Chagas disease, possibly involving its vector *Triatoma brasiliensis brasiliensis*, was identified in the state of Rio Grande do Norte (RN). Given the historical significance of this vector in public health, the study aimed to evaluate its role in the transmission dynamics of the protozoan *Trypanosoma cruzi* in an area undergoing desertification in the Seridó region, RN, Brazil. We captured triatomines in sylvatic and anthropic ecotopes. Natural vector infection was determined using parasitological and molecular methods and we identified discrete typing units (DTUs) of *T*. *cruzi* by analyzing the COII gene of mtDNA, 24Sα rDNA, and mini-exon gene. Their blood meals sources were identified by amplification and sequencing of the mtDNA cytochrome b gene. A total of 952 *T*. *b*. *brasiliensis* were captured in peridomestic (69.9%) and sylvatic ecotopes (30.4%). A wide range of natural infection rates were observed in peridomestic (36.0% - 71.1%) and sylvatic populations (28.6% - 100.0%). We observed the circulation of TcI and TcII DTUs with a predominance of Tcl in sylvatic and peridomestic environments. *Kerodon rupestris*, rocky cavy (13/39), *Homo sapiens*, human (8/39), and *Bos taurus*, ox (6/39) were the most frequently detected blood meals sources. Thus, *Triatoma b*. *brasiliensis* is invading and colonizing the human dwellings. Furthermore, high levels of natural infection, coupled with the detection of TcI and TcII DTUs, and also the detection of *K*. *rupestris* and *H*. *sapiens* as blood meals sources of infected *T*. *b*. *brasiliensis* indicate a risk of *T*. *cruzi* transmission to human populations in areas undergoing desertification.

## Author summary

Chagas disease currently affects about six to seven million people worldwide, resulting in high morbidity, mortality, and economic burden in endemic countries of Latin America. Its etiological agent, *Trypanosoma cruzi*, circulates among a wide variety of mammalian and insect vectors. *Triatoma brasiliensis brasiliensis* is adapted to the dry and warm climate of the Caatinga biome, and is considered the main vector in the semi-arid areas of northeastern Brazil. Information on the infestation, natural infection rates, *T*. *cruzi* strains, and blood meals sources of this vector is crucial for understanding the dynamics of *T*. *cruzi* transmission in areas susceptible to desertification. *Triatoma b*. *brasiliensis* colonizes peridomestic structures, particularly in the stone walls of cattle corrals that emerge as a refuge for sylvatic populations where they access a variety of blood meals sources. The predominance of the TcI strain in the sylvatic and peridomestic environments shows an overlap of transmission cycles by *T*. *cruzi* mediated by *T*. *b*. *brasiliensis*. The high rates of natural infection and the evidence of their feeding on humans and the rodent *K*. *rupestris* are worrisome and indicate the threat this vector poses to human health in the area studied.

## Introduction

Chagas disease currently affects approximately 6 to 7 million people globally and presents a high morbidity and mortality rate in endemic countries [1]. This disease produces different regional epidemiological patterns and is considered a serious public health problem in Latin America [1,2], a region recognized for high diversity of triatomines (Hemiptera: Reduviidae) [3,4], insect vectors of the etiological agent *Trypanosoma cruzi*. In this area, vertebrate hosts of the parasite, including reservoir hosts, involved in the transmission cycles of discrete typing units (DTUs) of *T*. *cruzi*, have been found in several biomes [5,6].

Species of triatomines of medical importance can invade and colonize human dwellings and establish a domestic *T*. *cruzi* cycle. The synanthropy of populations of some vector species has been increasing as a result of man-made degradation of natural biotopes, where the parasite circulates among mammals and sylvatic triatomines [7,8]. Considering its anthropozoonotic character, issues related to the persistence and spread of the Chagas disease, especially in areas with low socioeconomic development, intensive environmental degradation in the context of climate change, are among the main concerns of health authorities [1,9,10].

In semi-arid areas of northeastern Brazil, *Triatoma brasiliensis brasiliensis* is one of the main native vectors with domiciled populations known to transmit *T*. *cruzi* to humans [11–13]. This subspecies is included in the *Triatoma brasiliensis* species complex, monophyletic group [14] based on studies on morphology [15], biology [16], ecology, [17,18], experimental crosses [19,20], isoenzymes [21], and DNA analyses [22]. Currently, the complex is formed by six species and two subspecies [23–26], which can be identified based on taxonomic keys [14,27]. The taxa of this complex show differences regarding its epidemiological importance, based on biological, ecological, and behavioral parameters [17,28–30].

*Triatoma b*. *brasiliensis* is adapted to dry and hot environments, characteristic of the Caatinga biome, an eco-region suggested as its origin and source of dispersion [7]. Currently, this vector is found in the states of Ceará, Maranhão, Paraíba, Piauí, and Rio Grande do Norte [24], with high probability of expansion of its dispersal area due to advance of desertification [7], and climate change [31], which could thus imply the emergence or re-emergence of Chagas disease.

The remarkable biological plasticity of *T*. *b*. *brasiliensis* combined with its genetic variability allows for a variety of ecological relationships, resulting in the encroachment into the zoonotic cycles of the protozoan *T*. *cruzi* in the human population. Parasite genotyping studies have shown that this vector hosts TcI, TcII, and TcIII DTUs [32–35]. In addition, the DNA of other trypanosomatids such as *Trypanosoma rangeli*, which infects humans but is not pathogenic [36,37], was found in the intestines of *T*. *b*. *brasiliensis* [38,39]. Unlike several species of the genus *Rhodnius* [40,41], the vectorial competence of this subspecies in the transmission of that parasite to mammalian hosts has not yet been clarified. *Triatoma b*. *brasiliensis* presents varied and apparently increasing rates of natural infection by *T*. *cruzi* and is efficient in infesting the interior of homes, structures of the peridomestic setting, rocky outcrops [11,17,42,43], and the cactus *Pilosocereus gounellei* [44] in the sylvatic environment. Under severe drought conditions, this vector presents higher infestations in rocky outcrops and in human dwellings, which are considered high quality habitats [45]. Furthermore, *T*. *b*. *brasiliensis* is able to maintain colonies near human dwellings by feeding on a variety of domestic and peridomestic animals, whereas in the sylvatic environment, it feeds primarily on rodent species [17,35,46,47].

In the Seridó region, Rio Grande do Norte state, an area undergoing desertification, sylvatic populations of *T*. *b*. *brasiliensis* captured in a conserved area showed high rates of natural infection by *T*. *cruzi*, with the rodent *Kerodon rupestris* likely serving as a reservoir host [48]. Desertification, land degradation in low humidity areas have been observed since the 1970s, resulting from natural and/or anthropogenic factors [49]. In Seridó, deforestation of the Caatinga has resulted in increased agriculture, cattle raising, and the use of wood to produce firewood and coal, and surface mining which have further contributed to desertification. This phenomenon has led to the production of areas with degraded soils with high levels of salinization, resulting in soil infertility, erosion, and significant loss of biodiversity or migration of local fauna to stable environments, as well as an impact on human health [50,51].

In the context of Chagas disease, the migration of *T*. *b*. *brasiliensis* from sylvatic ecotopes to domiciliary ecotopes represents a potential risk of transmission of *T*. *cruzi* to humans. Invasion and/or colonization of this vector at home may result in infections by the classical vectorial pathway and/or by ingestion of food contaminated with feces [13]. Therefore, to better understand the transmission dynamics of *T*. *cruzi* in Caicó, we evaluated its infestation in natural and anthropologically altered ecotopes. The rates of natural infection by *T*. *cruzi*, DTUs of *T*. *cruzi* and blood meals sources were identified. This study provides specific information to support control and surveillance of Chagas disease vectors in this area.

## Material and methods

### Triatomines study area and capture

**Fig 1:**
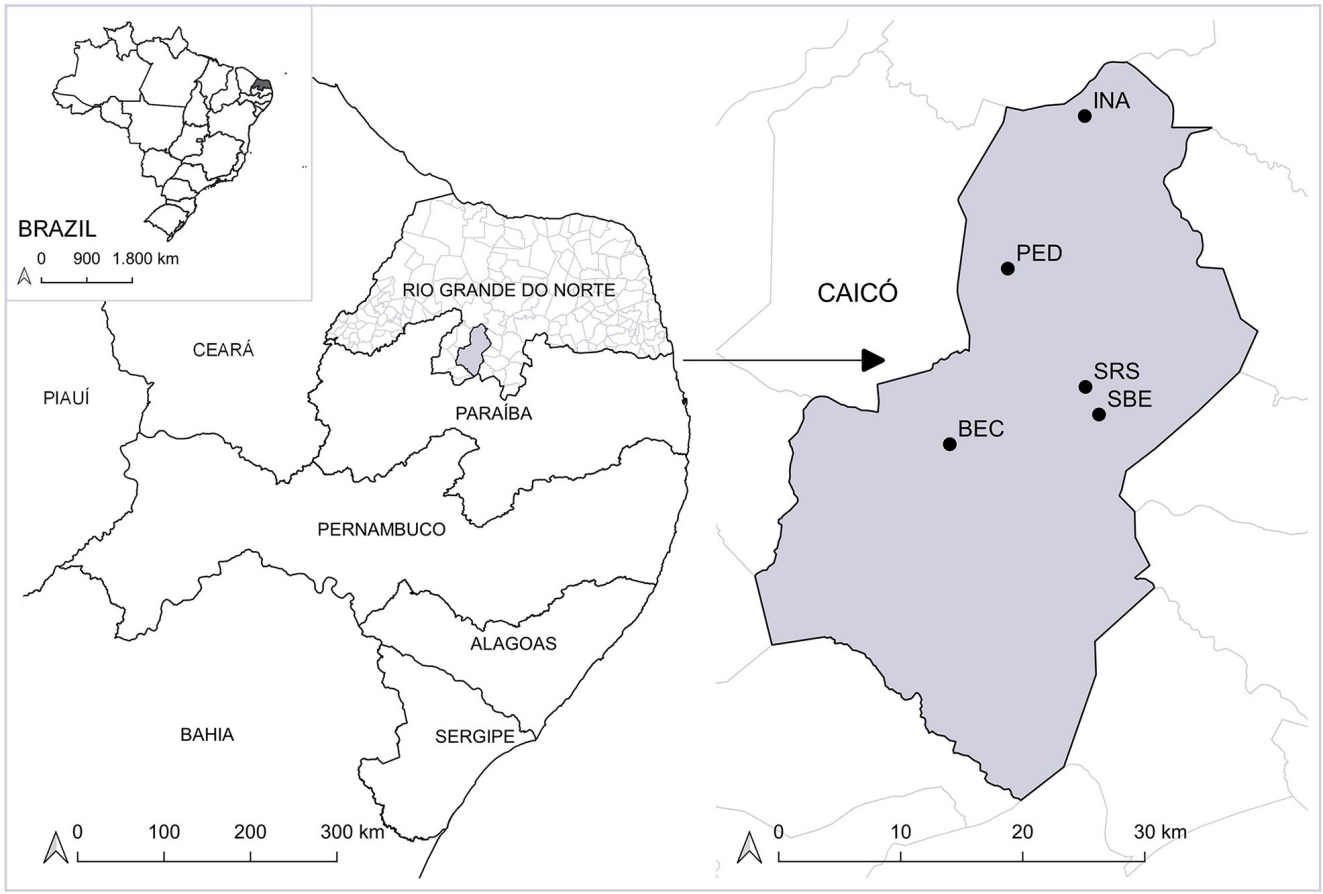
Geographical localization of collecting sites where triatomines were captured in Caicó municipality, Rio Grande do Norte, Brazil. The Gray map shows the limit of the Caicó municipality. Black dots indicate the small rural properties: Pedregulho (PED), Inácio (INA), São Bernardo de Elias (SBE), Riacho do Santo (SRS) and the Batalhão de Engenharia de Construção (BEC). This map was created using QGIS 3.4 software and cartographic bases obtained from the Brazilian Institute of Geography and Statistics (https://ibge.gov.br/).

This study was carried out in the municipality of Caicó (06°27’30”S 37°05’52”W), located in the Seridó Potiguar region, state of Rio Grande do Norte, Brazil, as shown below in the map (Fig 1). The map was constructed using Quantum GIS software version 3.4 (Madeira), using cartographic bases of the Brazil, Rio Grande do Norte, and Rio Grande do Norte municipality obtained from the Brazilian Institute of Geography and Statistics (IBGE). The municipality has a semi-arid climate, a high evaporation rate, and weak winds. Its climate is considered one of the driest and hottest in northeastern Brazil characterized by irregular rainfall, periods of drought ranging from 9 to 11 months, and concentrated rains in the summer and autumn [52]. Caicó is a part of the Caatinga biome, formed by a mosaic-pattern vegetation composed of medium and small species, thorny shrubs, and sparse xerophilous plants, in addition to rock formations.

The entomological research was carried out in 4 small rural properties: Pedregulho, Inácio, São Bernardo de Elias, and Riacho do Santo, and in one urban area, the Batalhão de Engenharia de Construção (Fig 1). We obtained permission for capture and transport of triatomines from Instituto Chico Mendes de Conservação da Biodiversidade/Ministério do Meio Ambiente (ICMBio-Sisbio, number 43393–1). Active searches were performed in domestic settings, defined as spaces closed by doors, comprising houses occupied by mainly humans. At each site, we inspected cracks in walls, areas behind furniture, objects leaning against the walls, cardboard under mattresses, and spaces under the beds. Entomological research was carried out during the day and night in the peridomestic (P) environment (area within 300m radius of each house). We examined henhouses, pigsties, cattle corrals, goats and sheep pens, piles of tiles, stones, wood, and rocky outcrops. Following the terminology used by Almeida et al. [48], we consider sylvatic areas divided into “Syl-d”–degraded areas–where humans, domestic animals and their vestiges (feces) can be found–and “Syl-c”–for conserved areas. There are species of fauna and flora preserved in these areas since access to humans is restricted and hunting of sylvatic animals is prohibited. Nocturnal active captures were carried out in rocky outcrops. The field activities had the collaboration of municipal and state official technicians working in the control of endemic diseases.

### Laboratory processing

Triatomines were identified based on their sex, instar, and species based on taxonomic keys [3,14,27]. To detect *T*. *cruzi* and identify the blood meals sources, the fourth, fifth instar nymphs, and adult insects were randomly chosen from each colony. These nymphal instars of Triatominae have more epidemiological importance because they have a more feed rate, infection probability, and dispersion capacity. After the extraction of the midgut and hindgut or faeces and urine drops collection under sterile conditions, the intestinal content was macerated with phosphate-buffered saline solution (1 M, pH = 7.2–7.4), 5-fluorocytosine (antifungal substance), and Penicillin-Streptomycin (antibiotic) (P4333, Sigma, São Paulo, Brazil) [53]. From this suspension of intestinal contents, aliquots were used for the following analyses: (1) identification of flagellated forms by light microscopy to infer the transmission of *T*. *cruzi* to vertebrates; (2) molecular identification of the protozoan *T*. *cruzi* using PCR, a more sensitive and species-specific method; (3) isolation of the parasite on culture medium for later identification of DTUs; and (4) identification of host blood meals.

### Molecular identification of *T*. *cruzi*

Total DNA was extracted from intestinal content samples (200μL aliquot). The extraction was carried out using IIlustra BloodGenomicPrep Mini spin kit (GE Healthcare Life Science, Chicago, USA), following the manufacturer’s instructions. In a fraction of samples, the phenol-chloroform method [54] was used. The pellets obtained were washed with 70% ethanol and resuspended in 30 μL ultrapure H_2_O. The purity was checked through absorbance measurement at 260 nm using a spectrophotometer (Denovix-Uniscience). When necessary, samples were dissolved to get a final DNA concentration of 20–50 ng/μL.

Molecular identification of *T*. *cruzi* was performed by amplification of the hypervariable regions of the minicircles of kinetoplast DNA, corresponding to a fragment of 330bp, by polymerase chain reaction (PCR), using primers 121 and 122 [55]. Since these primers also amplify the *T*. *rangeli* (300pb, 360pb and 760pb band profile), we used a positive control for this parasite [56]. In the same assay, in a multiplex format, we added the primers P2B and P6R, to amplify a fragment of 163bp referring to the 12S region of the ribosomal RNA of triatomines. Amplification of the triatomine gene served as a control for DNA extraction [57].

PCR was performed in a final volume of 25 μL, containing 2.5 μL of 10X Taq Buffer (Thermo Scientific, Massachusetts, USA), 2.5 μL of dNTPs (2 mM, Thermo Scientific, Massachusetts, USA), 4.5 μL of MgCl2 (25 mM, Thermo Scientific, Massachusetts, USA), 1 μL of each initiator 121 and 122 (10 pmol/μL), 0.3 μL of each initiator P2B and P6R (10 pmol/μL), 0.2 μL of Taq DNA polymerase (Stock solution: 5 U/μL) (Thermo Scientific, Massachusetts, USA), 7.7 μL of ultrapure H_2_O and 5 μL of DNA template. Positive controls used were 5 μL DNA (2 ng/μL) of acellular culture of *T*. *cruzi* strain Y and 5 μL DNA (2 ng/μL) of acellular culture of *T*. *rangeli* of the Macias strain; the same mixture of the reaction without DNA was used for the negative control. PCR was performed on the MaxyGene (Axygen) equipment according to the following thermal profile [55]. The amplified product was observed on a 3.5% agarose gel with 0.04% ethidium bromide under ultraviolet light.

### Identification of *T*. *cruzi* DTUs

Parasite isolation was performed on Novy-McNeal-Nicolle (NNN) and liver infusion tryptose (LIT) medium, and 500 μL of intestinal content was inoculated. Then, 10 μL of the culture was examined by light microscopy once a week for five months. The positive cultures for trypanosomatids had parasites cultivated on the LIT until the logarithmic phase. An aliquot of this culture was cryopreserved and maintained at the Laboratório Interdisciplinar de Vigilância Entomológica em Diptera e Hemiptera (IOC/FIOCRUZ, Rio de Janeiro, Brazil), and another aliquot was used for DNA extraction.

For DNA extraction and *T*. *cruzi* genotyping, the isolates were washed in PBS (1 M, pH = 7.2–7.4) by centrifugation at 2,500 rpm for 15 min at 4°C. After the third procedure, the pellet was resuspended in 300 μL ultrapure H_2_O and stored at -20°C until DNA extraction. Genomic DNA was extracted using the Illustra Blood GenomicPrep Mini Spin extraction kit (GE Healthcare Life Science, Chicago, USA), according to the manufacturer’s protocol.

The molecular characterization of *T*. *cruzi* isolates in DTUs was performed according to the protocol proposed in another study [58]: (1) We first PCR-amplified the mitochondrial gene encoding the cytochrome oxidase subunit 2 (COII), using the primers Tcmit-10 and Tcmit-21 according to the following thermal profile [59]. Next, we analyzed the restriction fragment length polymorphism of the COII gene using AluI restriction endonuclease. This marker can distinguish the DTUs: mitochondrial haplotype A (TcI) and mitochondrial haplotype C (TcII) from mitochondrial haplotype B (TcIII-VI). (2) Then, we amplified the divergent domain of the 24Sα gene of ribosomal DNA (24SαrDNA) using the primers D71 and D72 according to the following thermal profile [60]. Next, we did a PCR analysis of the spliced leader intergenic region (SL-IR) gene, using the TcIII and UTCC primers according to the following thermal profile [61] to distinguish the populations belonging to TcIII of TcI, TcII, and hybrid strains. We used samples of each genotype of *T*. *cruzi* as positive controls and the same reaction mixture without DNA as negative controls in all the reactions. The amplified product was observed on a 3.5% agarose gel with 0.04% ethidium [58] bromide under ultraviolet light.

### Identification of blood meals sources

Blood meals sources were identified as described by Dias et al. [62]. We performed PCR of the cytochrome b gene of mitochondrial DNA using universal primers for vertebrate animals named L14841 and H15149 [63]. These primers amplify a product of 307pb (excluding primers) without amplifying the DNA of the triatomine present in the tissue, which makes up the internal organs of the abdomen [64].

PCR was performed in a final volume of 50 μL with the following reagents: 5 μL of 10X Taq Buffer (Thermo Scientific, Massachusetts, USA), 5 μL dNTPs (0.2mM, Thermo Scientific, Massachusetts, USA), 4 μL MgCl_2_ (25 mM, Thermo Scientific, Massachusetts, USA), 1 μL of each primer L14841 and H15149 (10 pmol/μL), 0.3 μL of Taq DNA polymerase (5 U/μL, Thermo Scientific, Massachusetts, USA), 28.7 μL ultrapure H_2_O, and 5 μL of DNA template. For positive controls, 5 μL DNA (20ng/μL) of pig was added to the reaction mixture, and for the negative control, the same reaction mixture was used without DNA. PCR was performed on the MaxyGene (Axygen) instrument according to the following thermal profile [62]. The amplified product was observed on a 1.5% agarose gel with 0.04% ethidium bromide under ultraviolet light.

The amplified product samples were purified using the manufacturer’s Illustra GFX PCR DNA and Gel Band Purification kit (GE Healthcare Life Science, Chicago, USA), following the manufacturer’s instructions. For sequencing, BigDye Terminator v.3.1 Cycle Sequencing Kit was used in a DNA analyzer (ABI 3730), both from Applied Biosystems, according to Otto et al. [65]. The sequences obtained were edited using the BioEdit Sequence Alignment Editor 7.0.4.1 and [66] Mega7 programs, version 7.0.26 [67], and compared with the sequences deposited in the GenBank database using the BLAST tool. All the stages of this study followed the best Biosafety policies and practices.

## Results

### Triatomines infestation

**Fig 2:**
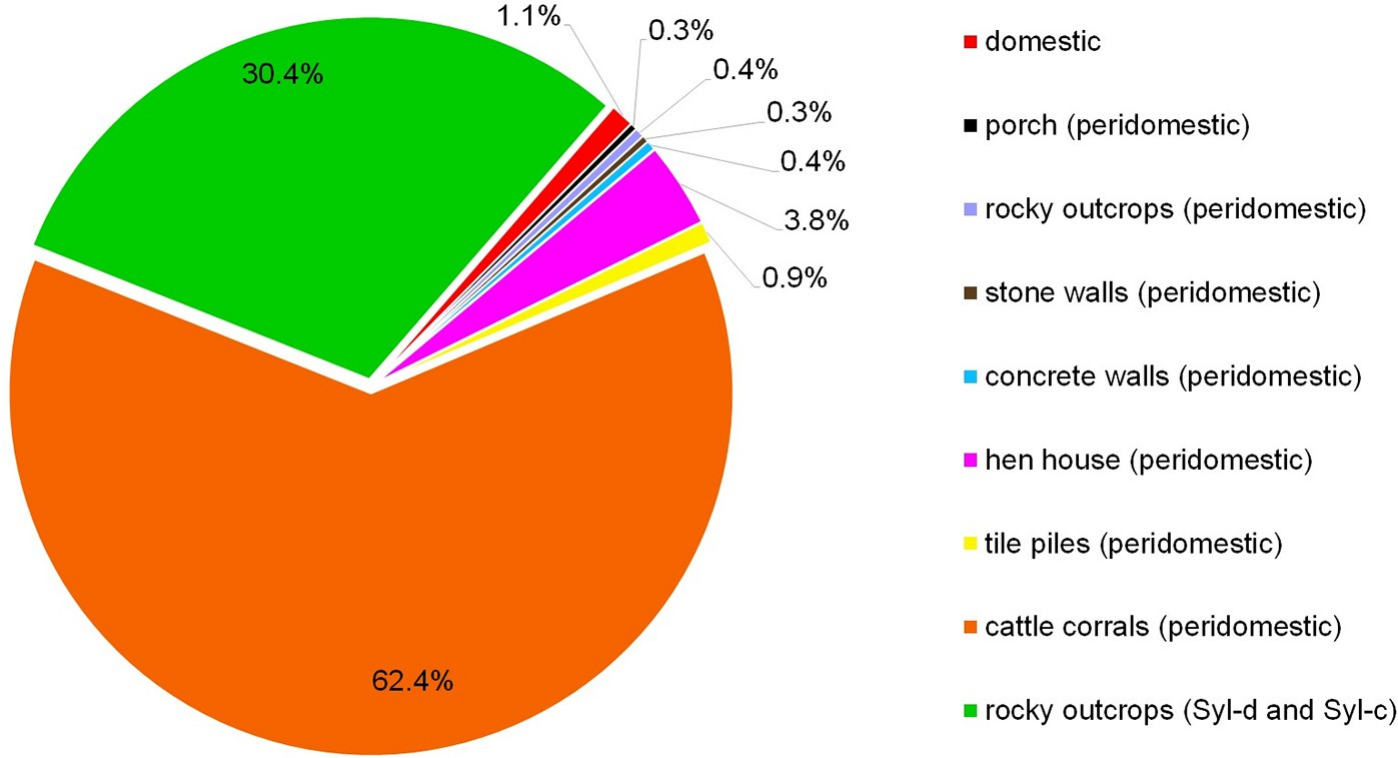
*Triatoma b*. *brasiliensis* infestation in the peridomestic and sylvatic ecotopes from Pedregulho, Inácio, São Bernardo de Elias, Riacho do Santo, and Batalhão de Engenharia de Construção localities in the Caicó municipality, Rio Grande do Norte, Brazil.

A total of 952 *T*. *b*. *brasiliensis* and 2 *Triatoma petrocchiae* were collected. *Triatoma b*. *brasiliensis* was found in all localities (Table 1), while *T*. *petrocchiae* was only collected at Inácio (two males, one in a domestic environment and the other in the sylvatic-d). The results showed a higher percentage of nymph stages 66.6% (634/952) than adults 33.4% (318/952), mainly in the peridomestic environment (Tables 1 and 2). Inside the houses, the percentages were equal (N = 50.0%, Ad = 50.0%) (Table 2). Among the ecotopes searched in the peridomestic setting, intense colonization of *T*. *b*. *brasiliensis* was observed in cattle corral 62.4% (594/952) (Fig 2).

**Table 1:** Number of *Triatoma b*. *brasiliensis* by locality, environment and life stage captured in the Caicó municipality, Rio Grande do Norte, Brazil.

Origin		Life Stage									
Locality	Environment	N1	N2	N3	N4	N5	M	F	N n (%)	Ad n (%)	Total n (%)
PED	Domestic	0	0	0	0	0	0	3	0 (0.0)	3 (100.0)	205 (21.5)
	Peridomestic	2	22	46	42	22	15	14	134 (82.2)	29 (17.8)	
	Syl-d	0	1	5	0	11	8	14	17 (43.6)	22 (56.4)	
Total		2	23	51	42	33	23	31	151 (73.7)	54 (26.3)	
SBE	Domestic	0	0	0	0	0	0	0	0 (0.0)	0 (0.0)	100 (10.5)
	Peridomestic	2	3	5	13	15	17	8	38 (60.3)	25 (39.7)	
	Syl-d	4	2	18	4	3	3	3	31 (83.8)	6 (16.2)	
Total		6	5	23	17	18	20	11	69 (69.0)	31 (31.0)	
INA	Domestic	0	0	0	2	3	2	0	5 (71.4)	2 (28.6)	211 (22.1)
	Peridomestic	0	2	32	30	57	33	22	121 (68.8)	55 (31.3)	
	Syl-d	0	0	4	1	6	10	7	11 (39.3)	17 (60.7)	
Total		0	2	36	33	66	47	29	137 (64.3)	74 (35.2)	
SRS	Domestic	0	0	0	0	0	0	0	0 (0.0)	0 (0.0)	309 (32.4)
	Peridomestic	0	3	14	63	98	29	44	178 (70.9)	73 (29.1)	
	Syl-d	0	0	3	10	15	14	16	28 (48.3)	30 (51.7)	
Total		0	3	17	73	113	43	60	206 (66.7)	103 (33.3)	
BEC	Syl-c	1	7	9	23	31	29	27	71 (55.9)	56 (44.1)	127 (13.3)
Total		9	40	136	188	261	162	158	634 (66.6)	318 (33.4)	952 (100.0)

PED, Pedregulho; SBE, São Bernardo de Elias; INA, Inácio; SRS, Riacho do Santo; BEC, Batalhão de Engenharia de Construção; Syl-c, sylvatic environment identified as conserved; Syl-d, environment identified as degraded; N1, first stage nymph; N2, second stage nymph; N3, third stage nymph; N4, fourth stage nymph; N5, fifth stage nymph; M, adult male; F, adult female; N, nymphs all stages; Ad, adult both male and female; n, number

**Table 2:** Environment infestation and life stage of *Triatoma b*. *brasiliensis* captured in Pedregulho, Inácio, São Bernardo de Elias, Riacho do Santo, and Batalhão de Engenharia de Construção localities in the Caicó municipality, Rio Grande do Norte, Brazil.

Origin	Life Stage									
Environment	N1 n (%)	N2 n (%)	N3 n (%)	N4 n (%)	N5 n (%)	M n (%)	F n (%)	N n (%)	Ad n (%)	Total n (%)
Domestic	0 (0.0)	0 (0.0)	0 (0.0)	2 (20.0)	3 (30.0)	2 (20.0)	3 (30.0)	5 (50.0)	5 (50.0)	10 (1.1)
Peridomestic	4 (0.6)	30 (4.6)	97 (14.9)	148 (22.7)	192 (29.4)	94 (14.4)	88 (13.5)	471 (72.1)	182 (27.9)	653 (68.6)
Syl-d	4 (2.5)	3 (1.9)	30 (18,5)	15 (9.3)	35 (21.6)	35 (21.6)	40 (24.7)	87 (53.7)	75 (46.3)	162 (17.0)
Syl-c	1 (0.8)	7 (5.5)	9 (7,1)	23 (18.1)	31 (24.4)	29 (22.8)	27 (21.3)	71 (55.9)	56 (44.1)	127 (13.3)
Total	9 (0.9)	40 (4.2)	136 (14.3)	188 (19.7)	261 (27.4)	160 (16.8)	158 (16.6)	634 (66.6)	318 (33.4)	952 (100.0)

Syl-c, sylvatic environment identified as conserved; Syl-d, sylvatic environment identified as degraded; N1, first stage nymph; N2, second stage nymph; N3, third stage nymph; N4, fourth stage nymph; N5, fifth stage nymph; M, adult male; F, adult female; N, nymphs all stages; Ad, adult both male and female; n, number.

### Natural infection by *T*. *cruzi*

A total of 287 *T*. *b*. *brasiliensis* gut contents were examined by light microscopy, of which 103 displayed *T*. *cruzi-like* parasites, resulting in a natural infection rate of 35.9%. All localities presented insects infected with *T*. *cruzi-like* parasites, and the highest percentages were detected in Riacho do Santo (58.5%) and Batalhão de Engenharia de Construção (54.1%) (Table 3). Among the environments searched, similar rates of natural infection were found in the peridomestic and sylvatic-d environments of the localities: Riacho do Santo (P = 59.3%, Syl-d = 56.5%), and Pedregulho (P = 13.6%, Syl-d = 10.0%), except in São Bernardo de Elias (P = 26.9%, Syl-d = 0.0%) (Table 3).

**Table 3:** Origin, blood meals sources and natural infection by *T*. *cruzi*; using Light Microscopy, PCR and Culture; of *Triatoma b*. *brasiliensis* captured in peridomestic and sylvatic environments of the localities in the Caicó municipality, Rio Grande do Norte, Brazil.

Origin			Blood Meals Sources			Light Microscopy				PCR				Culture (NNN-LIT)			
Loc	Env	Eco	n	Species	Popular name	Life Stage		NI		Life Stage		NI		Life Stage		NI	
						N (p/e)	Ad (p/e)	n (%)	Tn (%)	N (p/e)	Ad (p/e)	n (%)	Tn (%)	N (p/e)	Ad (p/e)	n (%)	Tn (%)
PED	Dom																
	Per	cc	1	*Gallus gallus*	Chicken	2/24	4/20	44 (13.6)	64 (12.5)	2/8	10/25	33 (36.4)	50 (36.0)	1/8	1/2	10 (20.0)	20 (15.0)
			2	*M*. *musculus*	Mouse												
	Syl-d	ro	2	*G*. *spixii*	Common cavy	1/4	1/16	20 (10.0)		1/3	5/14	17 (35.3)		0/2	1/8	10 (10.0)	
SBE	Dom																
	Per	p					0/1	26 (26.9)	35 (20.0)		0/1	25 (36.0)	32 (34.4)			6 (16.6)	9 (11.1)
		tp								0/3	1/1						
		hh				2/6	2/12			1/4	4/8			0/2			
		cc	1	*B*. *taurus*	Ox	0/2	2/3			1/1	2/3			1/1	0/2		
		ro	1	*C*. *hircus*	Goat	0/1				0/2				0/1			
		sw	2	*H*. *sapiens*	Human		1/1				1/2						
	Syl-d	ro				0/3	0/6	9 (0.0)		0/1	2/6	7 (28.6)		0/0	0/3	3 (0.0)	
INA	Dom		1	*K*. *rupestris*	Rocky cavy	0/1		1 (0.0)	69 (29.4)	1/1		1 (100.0)	60 (61.7)	0/1		1 (0.0)	32 (53.1)
	Per	cc	5	*B*. *taurus*	Ox	3/32	5/16	48 (16.7)		12/23	12/24	47 (51.1)		3/14	0/1	15 (20.0)	
			1	*M*. *musculus*	Mouse												
			1	*H*. *sapiens*	Human												
	Syl-d	ro	1	*H*. *sapiens*	Human	4/6	8/14	20 (60.0)		4/4	8/8	12 (100.0)		2/3	12/13	16 (87.5)	
			2	*K*. *rupestris*	Rocky cavy												
SRS	Dom																
	Per	cc	4	*K*. *rupestris*	Rocky cavy	15/31	20/28	59 (59.3)	82 (58.5)	13/22	34/45	67 (71.1)	91 (72.5)	8/12	5/5	17 (76.5)	31 (74.2)
			1	*G*. *spixii*	Common cavy												
			1	*M*. *musculus*	Mouse												
			1	*H*. *sapiens*	Human												
	Syl-d	ro	4	*K*. *rupestris*	Rocky cavy	2/7	11/16	23 (56.5)		4/5	15/19	24 (79.2)		1/3	9/11	14 (71.4)	
			1	*P*. *maculata*	Marsupial												
			1	*H*. *sapiens*	Human												
BEC	Syl-c	ro	2	*K*. *rupestris*	Rocky cavy	6/9	14/28	37 (54.1)	37 (54.1)	8/9	25/38	47 (70.2)	47 (70.2)	5/6	3/4	10 (80.0)	10 (80.0)
			1	*G*. *spixii*	Common cavy												
			1	*F*. *catus*	Cat												
			2	*H*. *sapiens*	Human												
Total			39			35/126	68/161		287 (35.9)	47/86	118/194		280 (58.9)	21/53	31/49		102 (51.0)

Loc, locality; PED, Pedregulho; SBE, São Bernardo de Elias; INA, Inácio; SRS, Riacho do Santo; BEC, Batalhão de Engenharia de Construção; Env, Environment; Dom, domestic environment; Per, peridomestic environment; Syl-c, sylvatic environment identified as conserved; Syl-d, sylvatic environment identified as degraded; Eco, ecotope; cc, cattle corral; ro, rocky outcrops; p, porch; tp, tile piles, hh, hen house; sw stone walls; n, number; *M*, *Mus*; *G*, *Galea*; *B*, *Bos*; *H*, *Homo*; *K*, *Kerodon*; *P*, *Planigale*; *F*, Felis; N, nymphs all stages; Ad, adult both male and female; NI, natural infection; Tn, total number.

Of the 280 specimens examined by conventional multiplex PCR, the amplified fragment corresponding to 330bp was detected in 165 samples, resulting in a natural infection rate of 58.9%. The highest rates of natural infection were recorded in Riacho do Santo 72.5% (66/91), Batalhão de Engenharia de Construção 70.2% (33/47), and Inácio 61.7% (37/60). Higher rates of infected bugs were detected in sylvatic-d than in the peridomestic setting at Inácio (Syl-d = 100.0%, P = 51.1%). In the other localities, the rates were similar: Pedregulho (Syl-d = 36.4%, P = 35.6%) and São Bernardo de Elias (Syl-d = 28.6%, P = 36.0%), especially in Riacho do Santo (Syl-d = 79.2%, P = 71.1%), where high rates were observed in both environments (Table 3).

Among the 102 cultures that did not contaminate (by fungi or bacteria), 52 were positive 51.0% (52/102). The results showed that the areas Batalhão de Engenharia de Construção 80.0% (8/10), Riacho do Santo 74.2% (23/31), and Inácio 53.1% (17/32) presented the highest rates of natural infection by *T*. *cruzi*, with high prevalence in the peridomestic (76.5%) and sylvatic-d (71.4%) environments in Riacho do Santo and sylvatic-d environment (87.5%) in Inácio (Table 3).

### Identification of *T*. *cruzi* DTUs

**Fig 3:**
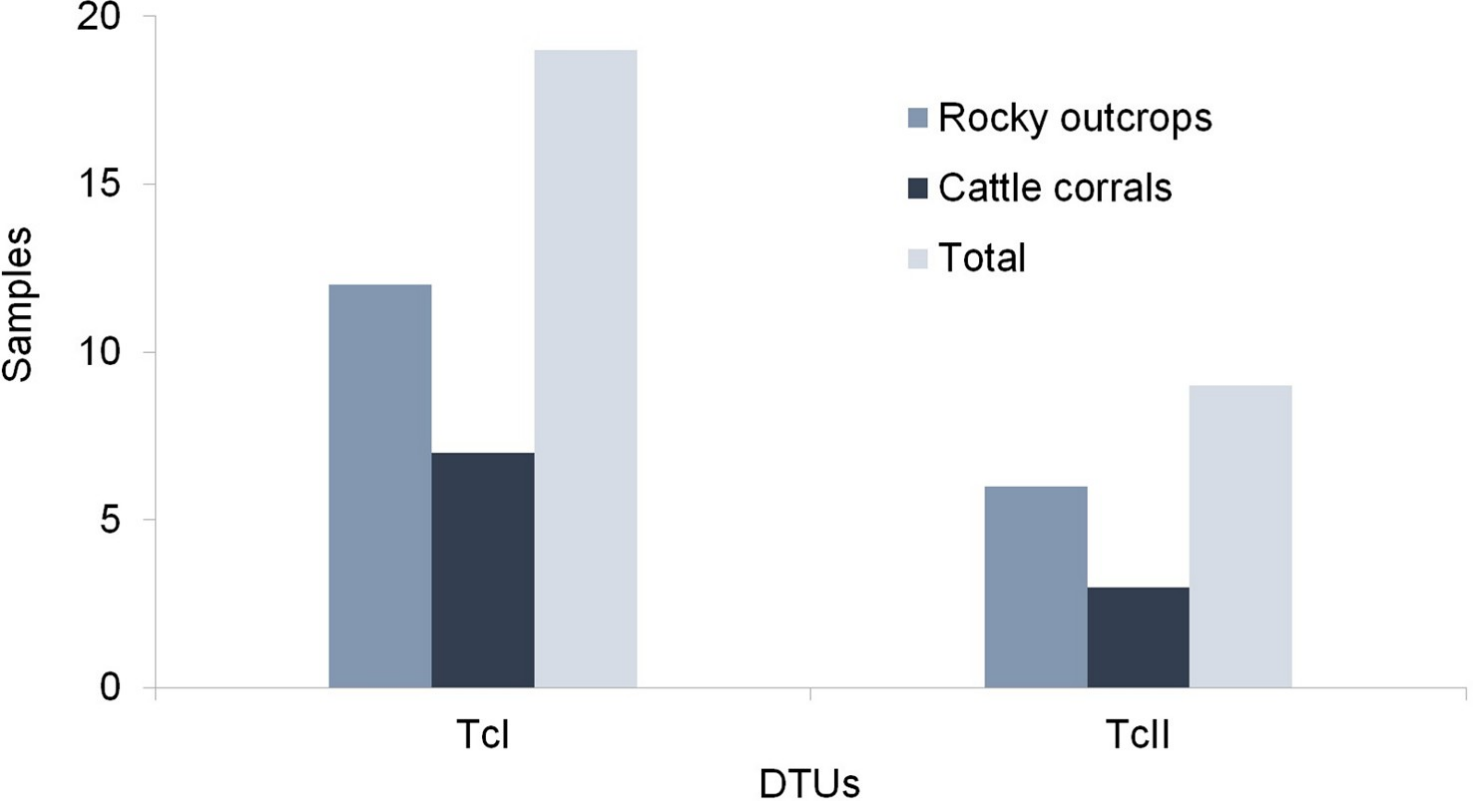
*Trypanosoma cruzi* DTUs distribution isolated of the *Triatoma b*. *brasiliensis* captured in the cattle corral (peridomestic setting) and rocky outcrops (sylvatic-d and sylvatic-c) from the Pedregulho, Inácio, São Bernardo de Elias, and Riacho do Santo and the Batalhão de Engenharia de Construção localities in the Caicó municipality, Rio Grande do Norte, Brazil.

A total of 28 *T*. *cruzi* isolates had parasite strains identified (Table 4). The circulating DTUs in populations of *T*. *b*. *brasiliensis* in the study area were TcI and TcII. The DTU detected at higher frequencies was TcI, 67.9% (19/28), present both in populations of the peridomestic (7 samples, cattle corral) and sylvatic-d and sylvatic-c environments (12 samples, rocky outcrop). The TcII DTU was identified at a lower frequency, 32.1% (9/28), but was distributed in rocky outcrops of sylvatic-d and sylvatic-c environments (6 samples) and the cattle corral in the peridomestic setting (3 samples) (Fig 3).

**Table 4:** List of *T*. *cruzi* DTUs isolated from *Triatoma b*. *brasiliensis* captured in the peridomestic and sylvatic environments of the localities in the Caicó municipality, Rio Grande do Norte, Brazil.

Origin			*T*. *b*. *brasiliensis*	*T*. *cruzi*				
Locality	Environment	Ecotope	Life Stage	COII—AluI	Haplotype	24Sα rDNA	SL-IR	DTU
PED	Peridomestic	cattle corral	N5	30bp 81bp 264bp	A	110bp	150bp	TcI
	Syl-d	rocky outcrops	Adult	30bp 81bp 264bp	A	110bp	150bp	TcI
SBE	Peridomestic	cattle corral	N5	82bp 212bp	C	125bp	150bp	TcII
INA	Peridomestic	cattle corral	N5	30bp 81bp 264bp	A	110bp	150bp	TcI
			N5	30bp 81bp 264bp	A	110bp	150bp	TcI
	Syl-d	rocky outcrops	Adult	30bp 81bp 264bp	A	110bp	150bp	TcI
			Adult	30bp 81bp 264bp	A	110bp	150bp	TcI
			Adult	30bp 81bp 264bp	A	110bp	150bp	TcI
			Adult	30bp 81bp 264bp	A	110bp	150bp	TcI
			N5	82bp 212bp	C	125bp	150bp	TcII
			N5	82bp 212bp	C	125bp	150bp	TcII
SRS	Peridomestic	cattle corral	N5	82bp 212bp	C	125bp	150bp	TcII
			Adult	30bp 81bp 264bp	A	110bp	150bp	TcI
			N5	30bp 81bp 264bp	A	110bp	150bp	TcI
			N5	30bp 81bp 264bp	A	110bp	150bp	TcI
			Adult	82bp 212bp	C	125bp	150bp	TcII
			N5	30bp 81bp 264bp	A	110bp	150bp	TcI
	Syl-d	rocky outcrops	Adult	30bp 81bp 264bp	A	110bp	150bp	TcI
			Adult	30bp 81bp 264bp	A	110bp	150bp	TcI
			N5	30bp 81bp 264bp	A	110bp	150bp	TcI
			Adult	30bp 81bp 264bp	A	110bp	150bp	TcI
			Adult	30bp 81bp 264bp	A	110bp	150bp	TcI
			Adult	82bp 212bp	C	125bp	150bp	TcII
BEC	Syl-d	rocky outcrops	Adult	30bp 81bp 264bp	A	110bp	150bp	TcI
			Adult	82bp 212bp	C	125bp	150bp	TcII
			N5	82bp 212bp	C	125bp	150bp	TcII
			Adult	30bp 81bp 264bp	A	110bp	150bp	TcI
			N5	82bp 212bp	C	125bp	150bp	TcII
Total								28

PED, Pedregulho; SBE, São Bernardo de Elias; INA, Inácio; SRS, Riacho do Santo; BEC, Batalhão de Engenharia de Construção; Syl-c, sylvatic environment identified as conserved; Syl-d, sylvatic environment identified as degraded; N5, fifth stage nymph; Adult, both male and female; bp, base pairs.

### Identified host blood meals

**Fig 4:**
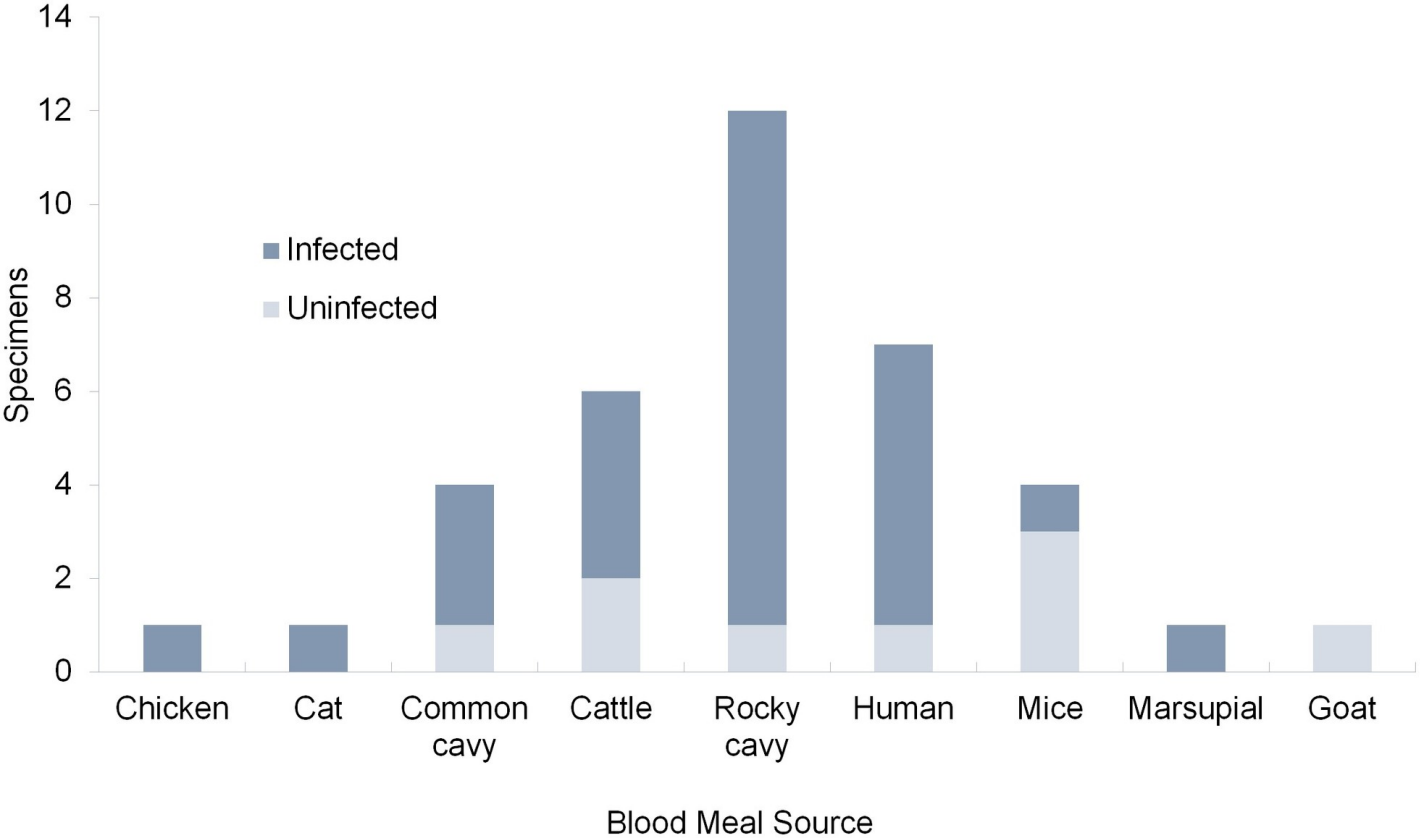
Blood meals sources of *Triatoma b*. *brasiliensis* identified in uninfected and infected specimens by *T*. *cruzi* from the Pedregulho, Inácio, São Bernardo de Elias, Riacho do Santo, and the Batalhão de Engenharia de Construção localities in the Caicó municipality, Rio Grande do Norte, Brazil.

The sequences obtained presented identity with the GenBank sequences that ranged from 89.42–99.72% (S1 File). The analysis of 39 intestinal contents samples allowed the identification of nine vertebrate species used as blood meals sources for the triatomines in the study area (Table 3). The most frequent hosts were *K*. *rupestris*, rock cavy 33.3% (13/39), *Homo sapiens*, human 20.5% (8/39), and *Bos taurus*, ox 15.4% (6/39) (Fig 4). Of the 39 specimens for which the blood meal source was identified, 28 were infected with *T*. *cruzi*, of which 35.7% (10/28) fed on the blood of *K*. *rupestris* and 21.4% (6/28) of *H*. *sapiens* (Fig 4).

**Fig 5:**
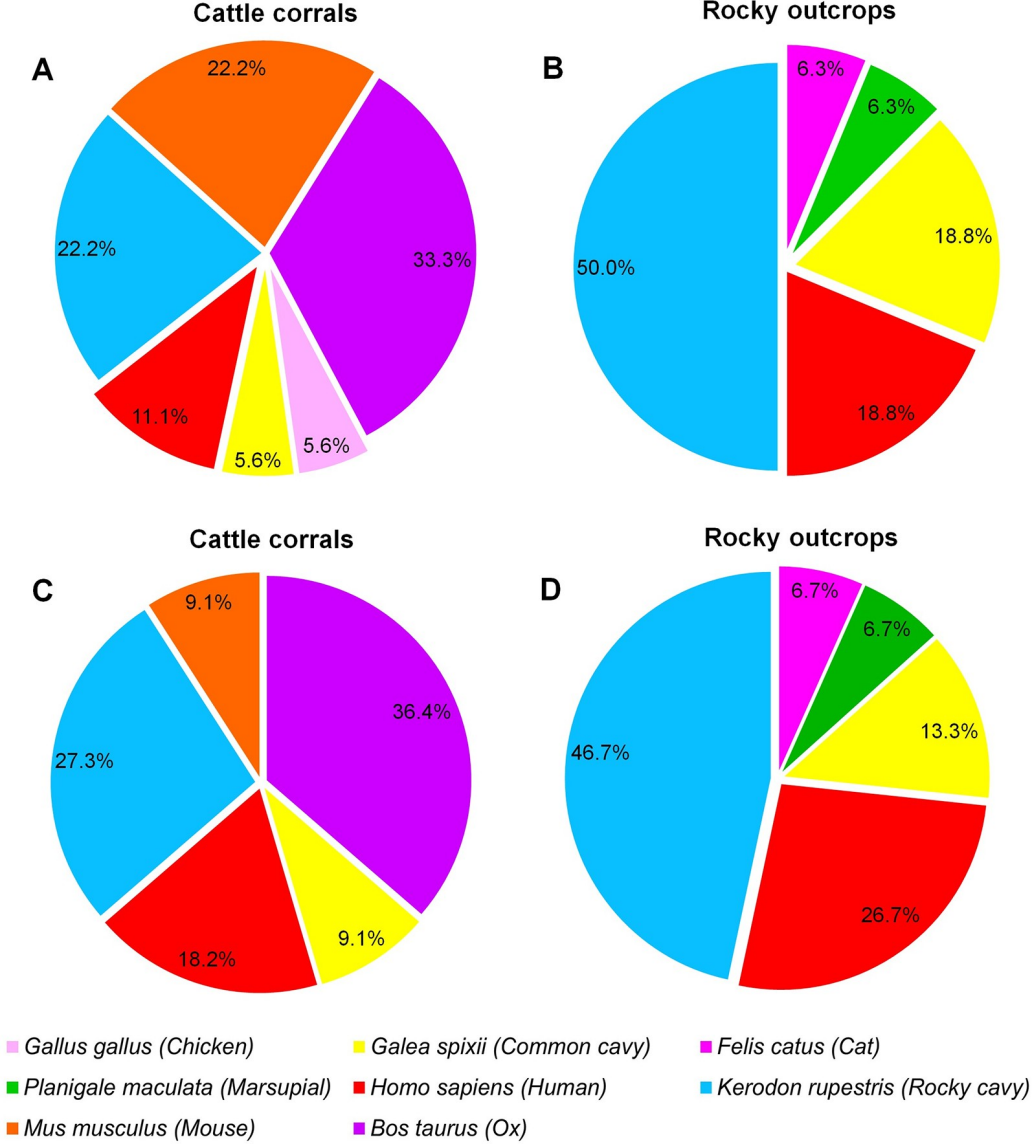
Blood meals sources of *Triatoma b*. *brasiliensis* identified in specimens from of cattle corral (peridomestic setting) and of rocky outcrops (sylvatic-d and sylvatic-c environments) from the Pedregulho, Inácio, São Bernardo de Elias, and Riacho do Santo and the Batalhão de Engenharia de Construção localities in the Caicó municipality, Rio Grande do Norte, Brazil. A-B. independent of *T*. *cruzi* positivity and C-D. of positives.

The type of blood meal source varied according to the environment. The blood of the sylvatic rodent *K*. *rupestris* was identified in specimens of *T*. *b*. *brasiliensis* in the peridomestic (4 specimens from the cattle corral) and in domestic settings (one specimen in an uninhabited house), in addition to the sylvatic-c and sylvatic-d environments. The blood of *Felis catus*, a domestic cat, was identified in the sylvatic-c environment specimen (in the rocky outcrop) (Table 3). In the peridomestic setting, the specimens captured in the cattle corral fed on a diversity of animals (6 species), with the highest percentage being that of *B*. *taurus*, 33.3% (6/18). However, we also found a considerable percentage of *K*. *rupestris* and *Mus musculus*, mouse, 22.2% (4/18) (Fig 5A). In the sylvatic-c and sylvatic-d environments, the rocky outcrops housed colonies whose specimens fed on five species of animals. Half of the blood meals sources identified were *K*. *rupestris* 50.0% (8/16), followed by *Galea spixii* (common cavy) and *H*. *sapiens*,18.8% (3/16) (Fig 5B). Among the infected insects, *B*. *taurus* 36.4% (4/11), *K*. *rupestris* 27.3% (3/11), and *H*. *sapiens* 18.2% (2/11) were the most frequent blood meals sources in cattle corrals (Fig 5C). In the rocky outcrops, *K*. *rupestris* 46.7% (7/15) and *H*. *sapiens* 26.7% (4/15) were the predominant blood meals sources (Fig 5D).

## Discussion

In this study, the characterization of the ecological relationships of *T*. *b*. *brasiliensis* collected from sylvatic and anthropogenic ecotopes, in areas experiencing a desertification process, indicate the risk of *T*. *cruzi* transmission to human populations. In addition, the data collected from the present study shed light on the underlying questions yet to be answered (i.e. What is the potential for transmission of *T*. *cruzi* from domestic animals to vectors? Could cattle act as a barrier to human infection?). Therefore, this study contributes to understanding the complexity of *T*. *cruzi* cycles and the risk factors implicated with human infections in these areas.

### Triatomines infestation

Our results showed that *T*. *b*. *brasiliensis* was predominant in degraded and natural ecotopes, as reported in other municipalities in Rio Grande do Norte [34,38]. We recorded this vector in domestic, peridomestic, and sylvatic environments, occupying nine diverse ecotopes. The high colonization capacity of *T*. *b*. *brasiliensis* populations in human dwellings was evidenced not only by the capture of immature forms inside houses, but also especially by the abundance of nymphs in peridomestic ecotopes, ratifying the results of other studies [34]. Among the habitats surveyed, stone walls of cattle corrals stood out as the most infested, possibly because it is a permanent peridomestic structure and often serves as a connection to the stone walls of the sylvatic environment, favoring the flow of triatomines and sylvatic rodents between the natural and degraded ecotopes of human dwellings.

The survival of the human population in areas experiencing desertification in Rio Grande do Norte is related to the constant adaptation to water scarcity. Thus, it is not uncommon to observe semi-extensive breeding of goats, sheep, and cattle in the Seridó region. In the dry season, cattle breeders confine the animals (mainly offspring) and complement their feeding with fodder mixed with water or the cactus *Opuntia cochenillifera* because of lack of water and native vegetation for pasture. In these circumstances, the presence of animals in the peridomestic setting may facilitate the triatomine-host interaction as we verified with cattle, which represented an important source of blood to maintain numerous colonies of *T*. *b*. *brasiliensis* in the vicinity of the human dwellings of the studied area.

### Natural infection by *T*. *cruzi*

The rates of natural infection by *T*. *cruzi* varied among the 5 localities where the populations of *T*. *b*. *brasiliensis* were sampled. Despite the differences in sensitivity, all three techniques indicated high rates at Inácio, Batalhão de Engenharia de Construção, and Riacho do Santo. It is important to note that specimens from these three localities fed on *K*. *rupestris*. Unlike the sites mentioned above, the populations from Pedregulho and São Bernardo de Elias had the lowest *T*. *cruzi* natural infection rates. These results possibly reflect the association of these populations with refractory hosts of this protozoan or hosts that are less competent in maintaining the parasite, such as chickens at Pedregulho and, possibly, cattle at São Bernardo de Elias and Inácio.

Curiously, while *Trypanosoma evansi* and *Trypanosoma vivax* infect and cause diseases in cattle [68,69], the same cannot be affirmed to *T*. *cruzi*. For that reason, we suggest further studies to investigate the role of these animals as refractory, sentinel, or reservoir hosts of *T*. *cruzi* within the study areas. The varied rates of natural infection show that within the same municipality, there are differences in the circulation of *T*. *cruzi*, which may be conditioned to the distribution of vertebrate species amplifiers of this protozoan. Therefore, the levels of *T*. *b*. *brasiliensis* infection may vary at different spatial and time scales. Different patterns of natural infection by *T*. *cruzi* have been observed among municipalities in the same state [34] and municipalities in different states [42]. The low rates of natural infection of sylvatic populations of *T*. *b*. *brasiliensis* at Pedregulho and São Bernardo de Elias concur with the results of a historic study [17]. In that study, the authors determined that 15.07% of the tested specimens were positive for *T*. cruzi using the fresh direct examination. Similarly, in a recent study [48], in which the same methodology was adopted, high rates of natural infection in populations from different localities were detected, ranging from 83% to 95%, in agreement with our results, where we found the highest rates in Riacho do Santo (58.5%) and Batalhão de Engenharia de Construção (54.1%).

The rates of natural infection were similar between peridomestic and sylvatic ecotopes in almost all studied localities. The analyses showed that only the peridomestic population (collected in the cattle corral) at Inácio showed a lower rate of natural infection by *T*. *cruzi*, although expressive, than its sylvatic-d conspecifics, which is in agreement with other studies [34]. Peridomestic setting natural infection levels may be related to blood repasts in host animals of *T*. *cruzi*, such as rodents *K*. *rupestris* at Inácio, where a population of this animal settled on a rocky outcrop near (20–50 m) the cattle corral. Other animals such as mice (*M*. *musculus*), at Pedregulho, and goats *(Capra hircus)* at São Bernardo de Elias were observed in the peridomestic setting and identified as a blood meal source of *T*. *b*. *brasiliensis* in the present study. Although the role of these animals in the peridomestic transmission cycle of *T*. *cruzi* is still being elucidated, studies have shown parasitemia [70] and exposure to infection in goats [71] and mice [72].

High rates of natural infection were detected in both environments at Riacho do Santo, which concurs with the results reported for the municipality of Currais Novos [42]. Although these specimens were captured in the cattle corrals, analyses of the gut content showed that the vectors fed on the blood of *K*. *rupestris* (4/7), *M*. *musculus* (1/7), and *G*. *spixii* (1/7). This evidence suggests an exchange of vectors between the sylvatic and peridomestic ecotopes. The high natural infection rate reported here reinforces the assumption that *K*. *rupestris* may be a potential reservoir of *T*. *cruzi* not only in preserved [48], but also in degraded areas. The density of small mammals is associated with the availability of water. Thus, prolonged periods of drought in areas susceptible to desertification are essential factors leading to the migration of rodents to peridomestic environments in search of more suitable habitats, where they can find habitats for protection, rest, and reproduction. In this context, the presence of rodents capable of maintaining parasitemia may explain the high rates of *T*. *cruzi* infection in the peridomestic ecotopes.

### Identification of *T*. *cruzi* DTUs

The frequency of *T*. *cruzi* DTUs in different environments, the peculiarities of their interactions with hosts, and their geographical distribution in the state of Rio Grande do Norte are still being investigated. Previous studies using the same methodology as the present study reported the occurrence of genetic strains TcI, TcII, and TcIII, both in the Potiguar west mesoregion and in the Potiguar central mesoregion of the state [32–34]. Our results showed the circulation of TcI and TcII DTUs at Inácio, Batalhão de Engenharia de Construção, and Riacho do Santo. At Pedregulho, we only identified Tcl DTU (2 samples), while at São Bernardo de Elias, we identified TcII (one sample), possibly because of the reduced number of samples analyzed at the latter localities. It is essential to highlight that these results refer to parasites isolated in culture medium; in other words, they were subjected to selective pressures, which may explain the low diversity among DTUs [73]. On the other hand, although we do not rule out the possibility of the presence of other DTUs in the study area, low diversity of *T*. *cruzi* strains can be expected in degraded areas, that is, areas presenting loss of mammal diversity, such as regions experiencing an intense desertification process [50]. A study conducted in Rio Grande do Norte suggested that TcII predominates in the domestic transmission cycle [32]. Another study indicated similar proportions of TcI (46.1%) and TcII (53.8%) in human samples [33]. In the present study, the results showed that TcI was more frequent than TcII in the peridomestic and sylvatic environments, this is additional evidence of an exchange between the sylvatic and peridomestic populations of *T*. *b*. *brasiliensis*, as observed in another study [35,74]. In areas of Caatinga, TcI and TcII DTUs have been identified in sylvatic hosts, such as *Didelphis albiventris* (TcI and TcII), *Rattus rattus* (TcI) [75], and *Trichomys laurentius* (TcI), as well as domestic mammals such as dogs (TcI) in Ceará [71].

### Identified host blood meals

The identification of nine vertebrate species showed the alimentary eclecticism of *T*. *b*. *brasiliensis*, which feeds on many hosts, confirming previous studies [17,35,46]. *Kerodon rupestris* was the primary feeding source of *T*. *b*. *brasiliensis* (infected) in rocky outcrops of sylvatic-d and sylvatic-c environments and an important blood source for populations of this vector occupying the peridomestic setting, reinforcing the possible role of this rodent as a reservoir host for *T*. *cruzi*. The present study revealed that triatomines (one specimen in an uninhabited house, 4 specimens in the cattle corral) fed on this animal were collected in anthropologically altered ecotopes. Of these 5 specimens that fed on *K*. *rupestris*, four were fifth instar nymphs, pointing the possibility of the presence of this rodent in the peridomestic setting since nymphs have limited dispersal capacity. This evidence is consistent with our field observations and with the reports of this sylvatic mammal getting closer to human dwellings.

In addition to the presence of *K*. *rupestris* in peridomestic areas, the data points to evidence of *T*. *b*. *brasiliensis*, especially adults, moving between domestic and sylvatic ecotopes. We identified human DNA in the intestinal contents of infected specimens from rocky outcrop, stone wall and cattle corral, indicating that after feeding in the domestic environment, the bugs return to their shelter in the sylvatic-d and peridomestic environments. This finding is a concern because it shows the transmission risk of the parasite to humans. In addition to *K*. *rupestris*, *G*. *spixii* was also identified as a blood source, although less frequently, in rocky outcrops 18,8% (3/16) and in cattle corral 5.6% (1/18). *Galea spixii* presents more pronounced synanthropic behavior than *K*. *rupestris*, which can also link the sylvatic and peridomestic cycles of *T*. *cruzi* in other areas [46].

Therefore, considering the acute scenario of environmental degradation in the Seridó region, we suggest longitudinal studies to survey the diversity of sylvatic mammals and their status as host or reservoir host of *T*. *cruzi* and the identification of the genotypes of this parasite in these animals. We can then confirm whether *K*. *rupestris* and *G*. *spixii* act as reservoir host for *T*. *cruzi* in this area.

## Conclusions

The colonization of *T*. *b*. *brasiliensis* was evidenced in the domestic and, especially in the peridomestic environments of human dwellings in the studied area, attesting to the persistence of the infestation of this vector in anthropologically altered ecotopes. This study provided evidence that the cattle corral serves as a refuge for this subspecies in the context of climate change in areas of intense desertification. Additionally, we recommend complementary research into the status of cattle as dispersers, sentinels, or barriers of *T*. *cruzi* transmission to the human population living in the study area. The circulation of TcI and TcII DTUs in the peridomestic and sylvatic-d and sylvatic-c environments, with a predominance of TcI in both environments, show the overlap of the sylvatic and peridomestic cycles of *T*. *cruzi* mediated by *T*. *b*. *brasiliensis*. The high rates of natural infection and evidence of feeding on humans and rodent *K*. *rupestris* is troubling and indicates a threat to humans residing in the study region. In this scenario, uninterrupted entomological surveillance activities associated with awareness-raising campaigns are necessary to avoid cases and outbreaks of Chagas disease in this region.

## Supporting information

S1 File Tb = *Triatoma b*. *brasiliensis*, RN = Rio Grande do Norte, SBE = São Bernardo de Elias, BEC = Batalhão de Engenharia de Construção de Caicó, Sylvatic-c = Sylvatic environment conserved, Sylvatic-d = Sylvatic environment degraded, N = Nymphal stages 1–5, Female = Adult Female, Male = Adult Male, Natural Infection_LM = Natural Infection by Light Microscopy, COII = cytocrome oxidase subunity 2, AluI = restriction endonuclease, 24SαrDNA = divergent domain of the 24SαrDNA, SL-IR = spliced leader intergenic region, bp = base pairs, DTU = discrete typing units. (XLSX)
